# Pilomatrix-Like High-Grade Endometrial Carcinoma (PiMHEC) With Secondary Vaginal Involvement: A Rare Variant of CTNNB1-Mutated Endometrial Carcinoma Mimicking Mesonephric-Like Adenocarcinoma

**DOI:** 10.7759/cureus.115658

**Published:** 2026-09-02

**Authors:** Ping Shi, Erik Washburn, Shaina F Bruce, Alice Dobi

**Affiliations:** 1 Department of Pathology, Penn State Health Milton S. Hershey Medical Center, Hershey, USA; 2 Department of Pathology, Penn State College of Medicine, Penn State Health Milton S. Hershey Medical Center, Hershey, USA; 3 Department of Obstetrics and Gynecology, Penn State College of Medicine, Penn State Health Milton S. Hershey Medical Center, Hershey, USA; 4 Department of Pathology and Laboratory Medicine, Penn State College of Medicine, Penn State Health Milton S. Hershey Medical Center, Hershey, USA

**Keywords:** cd10 marker, ctnnb1, endometrial carcinoma, mesonephric-like adenocarcinoma, pilomatrix-like carcinoma

## Abstract

Pilomatrix-like high-grade endometrial carcinoma (PiMHEC) is a recently recognized, clinically aggressive variant of endometrial carcinoma that is frequently misdiagnosed due to its morphological and immunophenotypic overlap with other high-grade endometrial malignancies. We report a 52-year-old woman who presented with widely metastatic high-grade carcinoma of endometrial origin, involving the vagina and distant sites including the brain, lung, and lymph nodes. Histologically, the tumor displayed a predominantly solid basaloid architecture with extensive geographic necrosis and abrupt keratinization with conspicuous shadow cells, supporting pilomatrix-like differentiation. Immunohistochemically, the tumor demonstrated strong, diffuse, aberrant nuclear β-catenin expression; patchy, heterogeneous expression of TTF1, GATA3 and CD10; and variable expression of CK7, CDX2, and p16. The tumor was negative for PAX8, ER, PR, and p40. p53 showed a wild-type pattern, and mismatch repair was retained (pMMR). Next-generation sequencing (NGS) identified a pathogenic exon 3 *CTNNB1* p.S33Ymutation. The morphologic, immunophenotypic, and molecular findings supported a diagnosis of PiMHEC. Notably, expression of TTF-1, GATA3, and CD10, together with absence of ER and PR expression, creates substantial immunophenotypic overlap with mesonephric-like adenocarcinoma (MLA) and represents a potential diagnostic pitfall. Recognition of characteristic shadow cell keratinization and aberrant nuclear β-catenin expression, integrated with molecular findings, is critical for distinguishing PiMHEC from MLA. This case expands the clinicopathologic spectrum of this underrecognized aggressive neoplasm and highlights the importance of integrating morphology, immunohistochemistry, and molecular findings to avoid diagnostic misclassification.

## Introduction

According to the fifth edition of the WHO Classification of Female Genital Tumours (2020), the principal histologic types of endometrial carcinoma include endometrioid carcinoma, serous carcinoma, clear cell carcinoma, undifferentiated carcinoma, dedifferentiated carcinoma, mixed carcinoma (composed of two or more distinct carcinoma types), and mesonephric-like adenocarcinoma (MLA) [1]. Pilomatrix-like high-grade endometrial carcinoma (PiMHEC) is a rare, recently recognized variant of endometrial carcinoma. Given the limited number of reported cases and the evolving understanding of this entity, it has not yet been included in the 2020 WHO Classification of Female Genital Tumours or incorporated into the FIGO 2023 staging system [2]. Histologically, PiMHEC is characterized by a solid basaloid architecture with extensive necrosis, abrupt keratinization and conspicuous shadow (“ghost”) cell formation, closely resembling cutaneous pilomatrix carcinoma [3]. At the immunohistochemical and molecular levels, PiMHEC is characterized by aberrant nuclear β-catenin expression and frequent exon 3 mutations in CTNNB1, implicating dysregulation of the Wnt/β-catenin pathway in its pathogenesis [4,5,6]. These tumors typically lack expression of PAX8, ER, PR, and p40. Consequently, the immunophenotypic profile of PiMHEC may pose a diagnostic challenge, particularly at extragenital or metastatic sites [7,8]. In addition, as illustrated by the present case, PiMHEC may express TTF-1, GATA3, and CD10, potentially creating substantial immunophenotypic overlap with MLA.

MLA is another rare subtype of gynecological carcinoma, most commonly arising in the endometrium and ovary, and is now included in the 5th edition of the WHO Classification of Female Genital Tumours. MLA is characterized by mesonephric differentiation and variable architectural patterns including solid, tubular, glandular, ductal, and papillary growth. Immunohistochemically, MLA typically demonstrates expression of TTF-1 and GATA3, luminal staining for CD10, absence of ER and PR expression, and variable PAX8 expression [9]. Molecularly, MLA is characterized predominantly by activating KRAS mutations and alterations involving the RAS/MAPK pathway, although rare tumors harboring CTNNB1 mutations have been reported [10]. Consequently, the overlapping morphologic and immunophenotypic features, together with occasional convergence in molecular alterations, may result in misclassification of PiMHEC as MLA.

Distinguishing PiMHEC from MLA is clinically relevant because these tumors represent biologically distinct entities with different characteristic molecular alterations and incompletely defined but potentially distinct clinical trajectories. Both demonstrate aggressive behavior and metastatic potential; however, accurate classification is essential for appropriate pathologic diagnosis, prognostic assessment, and interpretation of molecular findings, particularly as molecularly guided therapeutic strategies continue to evolve.

Here, we present a rare case of PiMHEC with secondary vaginal involvement and widespread metastases, demonstrating an unusual immunophenotypic overlap with MLA, including expression of TTF-1, GATA3, and CD10. Secondary vaginal involvement by PiMHEC has rarely been reported in the literature, and the present case further expands the limited clinicopathologic data regarding the patterns of spread of this aggressive neoplasm.

## Case presentation

A 52-year-old G2P2002 postmenopausal woman with a medical history of class III obesity and tobacco use initially presented with postmenopausal bleeding. Her gynecologic history was notable for menarche at age 9 and menopause at approximately age 49. Transvaginal ultrasonography demonstrated an enlarged uterus with endometrial thickening. An endometrial biopsy was attempted after her initial presentation but was unsuccessful because of cervical stenosis, and further outpatient evaluation of the uterine mass was planned. Approximately one month later, the patient developed progressive fatigue, lethargy, confusion, and new bilateral lower-extremity weakness. She was subsequently taken to the emergency department. Neurologic examination demonstrated marked bilateral lower-extremity weakness, with strength of at least 2/5. Abdominal examination revealed a soft, non-tender, non-distended abdomen without rebound or guarding. Given her acute neurologic symptoms and concern for a cerebrovascular event, computed tomography (CT) of the head was performed and demonstrated multiple discrete intracranial lesions, raising concern for metastatic malignancy. Subsequent brain magnetic resonance imaging (MRI) confirmed multiple intracranial metastases with surrounding vasogenic edema (Fig. 1A). Systemic imaging was subsequently performed to identify the primary tumor and assess the extent of disease. Pelvic imaging demonstrated an enlarged uterus with a large exophytic mass distending the endometrial cavity, with extension through the cervical canal into the vagina (Fig. 1B). Additional imaging findings consistent with disseminated disease included adnexal involvement, pulmonary nodules, and inguinal lymphadenopathy. Serum tumor markers showed a carcinoembryonic antigen (CEA) level of 8.8 ng/mL, cancer antigen 125 (CA-125) level of 25.7 U/mL, and carbohydrate antigen 19-9 (CA 19-9) level of 24.6 U/mL.

**Figure 1:**
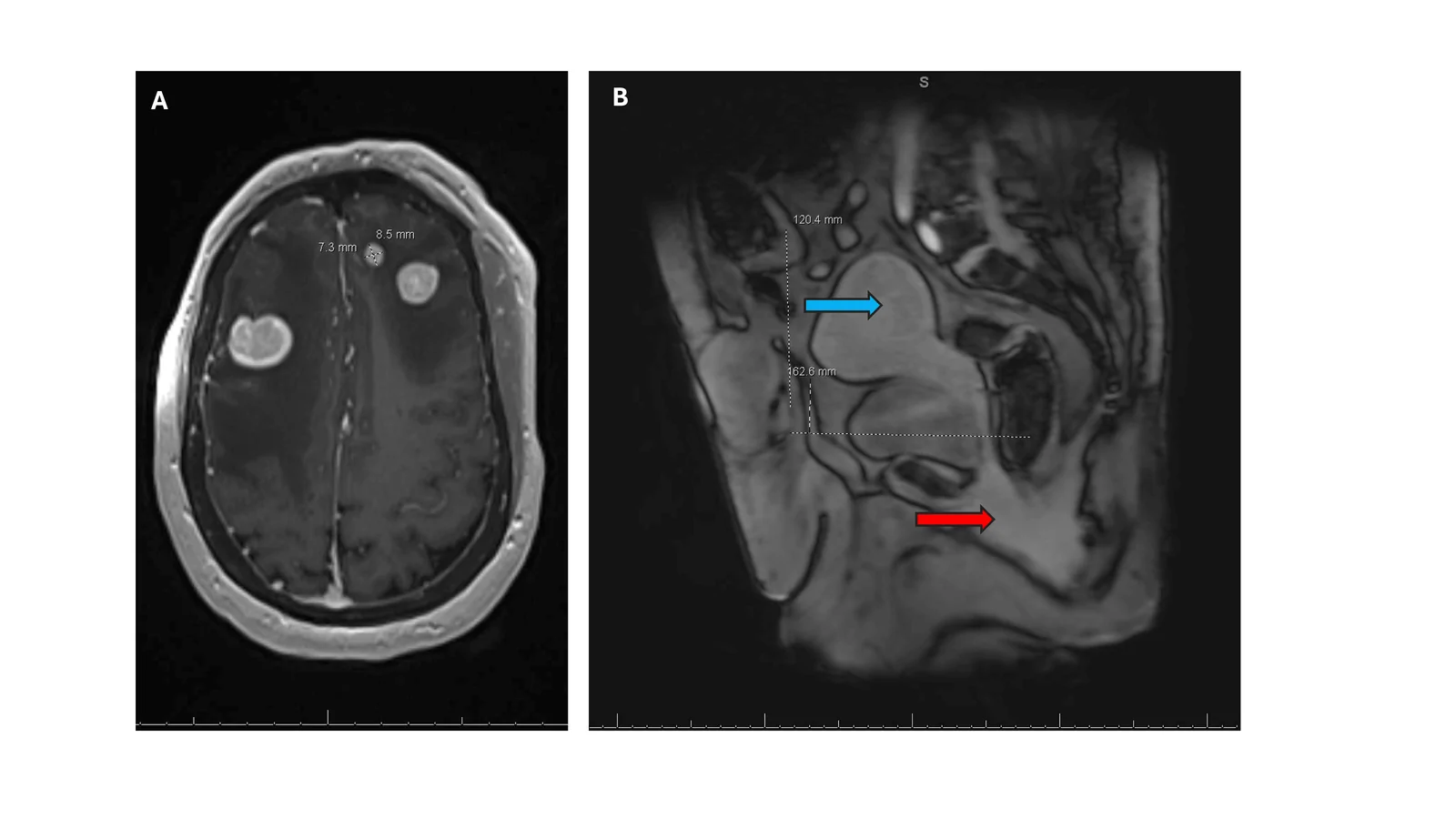
. Magnetic resonance imaging (MRI) findings. A. Contrast-enhanced axial brain MRI demonstrating multiple enhancing metastatic lesions in both cerebral hemispheres with surrounding vasogenic edema. B. Sagittal pelvic MRI demonstrating a large uterine mass occupying the endometrial cavity (blue arrow) with inferior extension into the cervix and vagina (red arrow).

The patient underwent hysteroscopy with dilation and curettage, partial vaginectomy, and excision of tumor involving the distal urethra. Histologic examination of the endometrial curettage revealed a predominantly high-grade carcinoma composed of solid nests and sheets of basaloid tumor cells with hyperchromatic nuclei, prominent nucleoli, extensive geographic necrosis, and abrupt keratinization with shadow (“ghost”) cell formation, comprising over 95% of the tumor volume (Fig. 2A). Minor foci (<5%) displayed low grade papillary endometrioid-type glands (Fig. 2B). Immunohistochemistry demonstrated strong, diffuse, aberrant nuclear β-catenin expression suggesting underlying CTNNB1 mutation (Fig. 2C). Tumor cells showed heterogeneous staining for CK7, CDX2 and p16 (approximately 50% of tumor cells) and were negative for PAX8, ER, PR and p40. Synaptophysin and INSM1 were focally positive in approximately 30% and 10% of tumor cells, respectively. p53 showed a wild-type pattern, and mismatch repair was retained (pMMR). The tumor also showed patchy, heterogeneous expression of TTF-1 and GATA3 (each involving approximately 50% of tumor cells with moderate staining intensity), as well as CD10. (Fig 2D-2F). Next-generation sequencing (NGS) identified pathogenic variants in CTNNB1 (exon 3, p.S33Y), ARID1A (exon 20, p.L2155fs), and PIK3CA (exon 2, p.E110del), as well as two PTEN alterations: a likely pathogenic in-frame deletion in exon 7 (p. G251del) and a pathogenic frameshift variant in exon 5 (p.P96fs). The vaginal mass mirrored the basaloid morphology of the uterine tumor, with no overt glandular differentiation. Surrounding benign squamous vaginal mucosa was present, supporting secondary vaginal involvement by PiMHEC.

**Figure 2:**
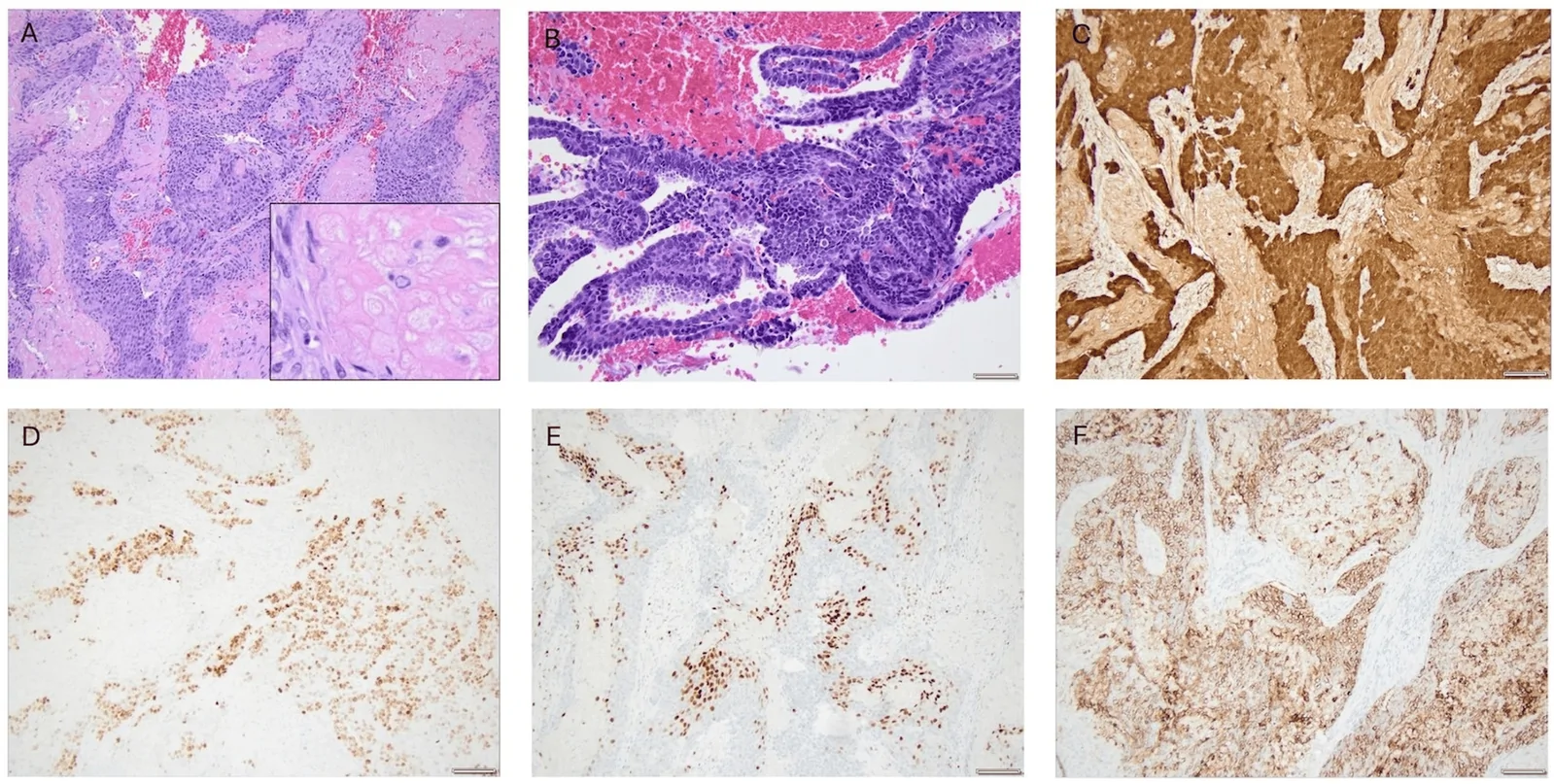
Histologic and immunohistochemical features of pilomatrix-like high-grade endometrial carcinoma (PiMHEC). A. High-grade solid basaloid component with geographic necrosis and abundant shadow (“ghost”) cells (H&E, 100×) and shadow (“ghost”) cells (H&E, inset, 600×); B. Minor low-grade endometrioid-type glandular component (H&E, 100×); C. Aberrant nuclear expression of β-catenin in high-grade tumor component (IHC, 100×); D. Patchy TTF-1 expression (IHC, 100×); E. Patchy GATA3 expression (IHC, 100×); F. Patchy cytoplasmic and membranous staining of CD10 (IHC, 100×). 100 µm bars in B-F.

The case was reviewed at a multidisciplinary tumor board, and the disease was clinically staged as FIGO stage IVB. The patient completed five fractions of whole-brain radiotherapy, followed by initiation of systemic chemotherapy with carboplatin and paclitaxel. Paclitaxel was discontinued because of an acute infusion reaction and was subsequently replaced with nab-paclitaxel. Pembrolizumab was later incorporated into the treatment regimen. After five cycles of carboplatin, nab-paclitaxel, and pembrolizumab, restaging CT demonstrated an interval decrease in the size of multiple pulmonary nodules and mediastinal, retroperitoneal, and pelvic lymphadenopathy without new sites of disease. A sixth cycle of systemic therapy was planned. At the time of manuscript preparation, eight months after initial diagnosis, the patient remained under active follow-up by the Gynecologic Oncology team.

## Discussion

PiMHEC is a rare, aggressive, and recently recognized variant of endometrial carcinoma characterized by a solid basaloid architecture, frequent geographic necrosis, and abrupt keratinization with shadow (“ghost”) cell formation [3,5,6]. Since its initial description by Weisman et al. in 2022, PiMHEC remains underrepresented in major classification systems, including the 2020 WHO Classification of Female Genital Tumours [1] and the 2023 FIGO staging system [2], likely due to its rarity and diagnostic challenges, particularly in limited samples and at metastatic sites.

This case highlights several key clinicopathologic features that support the diagnosis of PiMHEC and underscore its potential for misclassification. Morphologically, the tumor showed a predominantly basaloid component with extensive geographic necrosis, abrupt keratinization, and shadow cell formation (>95% of tumor volume) with only minor residual low-grade endometrioid-type glands (<5%), highly specific for PiMHEC and critical for distinguishing it from other high-grade uterine carcinomas [4].

Diffuse nuclear β-catenin staining and CTNNB1 mutation are hallmark features of PiMHEC. Additional features, including loss of PAX8, ER, PR, and p40 in the high-grade component, further cause the diagnostic difficulty, particularly in extragenital and metastatic sites where traditional Müllerian markers may be absent [7]. A major diagnostic pitfall in this case was the expression of TTF-1, GATA3, and CD10, markers commonly associated with MLA. However, the pattern of CD10 staining may provide a useful diagnostic clue: MLA typically demonstrates luminal staining, whereas PiMHEC exhibits cytoplasmic and membranous expression, as observed in the present case. Molecular findings may provide additional discriminatory information. Although KRAS mutations are characteristic of MLA, rare cases harboring CTNNB1 mutations have been reported [10], further highlighting the potential diagnostic overlap between MLA and PiMHEC.

MLA is generally regarded as a Müllerian neoplasm exhibiting mesonephric differentiation or mesonephric-like lineage reprogramming. In this setting, GATA3 likely reflects activation of a transcriptional activity associated with mesonephric differentiation; CD10 expression similarly supports mesonephric-type differentiation, particularly when localized to the luminal or apical surface of glands and tubules; and TTF1 may mark the same lineage shift. In contrast, in PiMHEC, expression of these markers may result from CTNNB1/β-catenin-driven lineage plasticity within a Müllerian neoplasm, leading to aberrant activation of overlapping differentiation programs. Accordingly, the shared immunophenotype may represent partial convergence on similar transcriptional pathways despite fundamentally distinct tumor identities.

Morphologically, MLA is composed of cuboidal to columnar cells with vesicular nuclei, nuclear grooves, and pale eosinophilic cytoplasm, typically arranged in tubular, ductal, papillary, or solid architectures. In contrast, the tumor in our case showed predominantly solid nests and sheets of basaloid cells with hyperchromatic nuclei, prominent nucleoli, and a geographic growth pattern, including abrupt keratinization, shadow (“ghost”) cell formation, and frequent geographic necrosis--hallmark features of PiMHEC (Table 1). Both PiMHEC and MLA are aggressive tumors with a propensity for distant metastasis. The aggressive course in this patient, including widespread metastases, is consistent with prior reports of PiMHEC's infiltrative nature [5]. The secondary vaginal involvement broadens the recognized clinicopathologic spectrum of PiMHEC and presents a potential diagnostic challenge in establishing the primary site.

**Table 1:** Pathologic comparison of pilomatrix-like high-grade endometrial carcinoma (PiMHEC) and mesonephric-like adenocarcinoma (MLA) Abbreviations: IHC: Immunohistochemistry; KRAS: KRAS proto-oncogene; CTNNB1: Catenin beta 1

Feature	PiMHEC	MLA
Location	Endometrium (most common) and ovary; often with an associated low-grade endometrioid component	Endometrium (most common) and ovary
Growth pattern	Predominantly solid basaloid nests/sheets	Heterogeneous: tubular/ductal, glandular, papillary, solid
Defining histologic clue	Abrupt keratinization with shadow (“ghost”) cells	No shadow cell keratinization
Necrosis	Often geographic and/or comedo-type	Variable; not defining
Cytologic features (typical)	Basaloid cells with hyperchromasia; often prominent nucleoli	Cuboidal–columnar cells; overlapping vesicular nuclei; nuclear grooves and marginated chromatin may be present
β-catenin (IHC)	Diffuse aberrant nuclear β-catenin (hallmark)	Typically not diffuse nuclear; may be variable in rare cases
CTNNB1	Common; usually exon 3 mutations (e.g., p.G34R)	Uncommon; rare cases reported
KRAS	Not characteristic	Common
TTF-1	May be patchy positive	Often positive
GATA3	May be patchy positive	Often positive
CD10	May be positive with cytoplasmic/membranous pattern	Often positive with luminal pattern

Given their rarity, no histology-specific standard systemic therapy has been established, and management generally follows treatment approaches for high-risk or advanced endometrial carcinoma, including surgery, chemotherapy, and radiotherapy. Both MMR-proficient and MMR-deficient PiMHEC cases have been reported [4,7,8,11]. Notably, a marked response to immune checkpoint inhibitor (CPI) therapy has been described in a patient with MMR-deficient PiMHEC [8], suggesting a potential role for immunotherapy in selected patients. However, given the rarity and recent recognition of PiMHEC, the efficacy of CPI therapy has not been systematically evaluated in this entity. In contrast, MLA is typically MMR proficient and frequently harbors KRAS mutations. Sustained responses to pembrolizumab plus lenvatinib have been reported in two patients with recurrent KRAS-mutated endometrial MLA [12]. Overall, further studies are needed to define optimal treatment strategies for the distinct molecular profiles of these rare tumors.

## Conclusions

This case highlights PiMHEC as a rare and aggressive tumor with significant morphologic and immunophenotypic overlap with MLA. Recognition of its distinctive histologic features-especially ghost cell keratinization-and its CTNNB1 mutation-driven profile is essential for accurate diagnosis. 

## References

[1] Kim KR, Lax SF, Lazar AJ (2020). Tumors of the uterine corpus. WHO Classification of Tumors, Female Genital Tumors (5th ed).

[2] Berek JS, Matias-Guiu X, Creutzberg C (2023). FIGO staging of endometrial cancer: 2023. J Gynecol Oncol.

[3] Weisman P, Park KJ, Xu J (2022). FIGO grade 3 endometrioid adenocarcinomas with diffusely aberrant β-catenin expression: An aggressive subset resembling cutaneous pilomatrix carcinomas. Int J Gynecol Pathol.

[4] Xu J, Park KJ, Weisman PS (2024). An expanded series of pilomatrix-like high-grade endometrioid carcinoma (Pimhec), including both MMR deficient and MMR proficient cases. Int J Gynecol Pathol.

[5] Zhou F, Qin L, Huang S, Lin W, Zhang H, Parkash V, Zheng W (2025). Pilomatrix-like high-grade endometrial carcinoma: An underdiagnosed entity with aggressive clinicopathologic features. Am J Surg Pathol.

[6] Buza N, Sun T, Hui P (2026). Endometrial carcinomas - Challenges and updates on selected topics. Hum Pathol.

[7] Arciuolo D, Travaglino A, Santoro A, Roberti P, Raffone A, Inzani F, Zannoni GF (2023). Pilomatrix-like high-grade endometrioid carcinoma is a morphologically and immunophenotypically distinct entity and may show mismatch repair deficiency. Int J Gynecol Pathol.

[8] Ajmal N, Nicolais O, Shahin MS, Ma L (2025). Pilomatrix-like high-grade endometrioid carcinoma of the uterine corpus with excellent response to immune checkpoint inhibitor therapy. Int J Gynecol Pathol.

[9] Kolin DL, Costigan DC, Dong F, Nucci MR, Howitt BE (2019). A combined morphologic and molecular approach to retrospectively identify KRAS-mutated mesonephric-like adenocarcinomas of the endometrium. Am J Surg Pathol.

[10] Kang B, Han B, Shen D, Zhang G, Zhang X (2025). Clinical and pathological analysis of 17 cases of mesonephric-like adenocarcinoma. Pathol Res Pract.

[11] Rusnell L, Al-Shamma Z, Bisch SP (2025). Uterine endometrioid adenocarcinoma with pilomatrix-like differentiation: A case report and review of the literature. Gynecol Oncol Rep.

[12] Shen S, Rubinstein MM, Park KJ, Konner JA, Makker V (2021). Sustained response to lenvatinib and pembrolizumab in two patients with KRAS-mutated endometrial mesonephric-like adenocarcinoma. Gynecol Oncol Rep.

